# PML-RARA mutations confer varying arsenic trioxide resistance

**DOI:** 10.1007/s13238-016-0356-4

**Published:** 2016-12-27

**Authors:** Dong-Mei Bai, Xiao-Feng Zheng

**Affiliations:** 1 State Key Lab of Protein and Plant Gene Research, Beijing, 100871 China; 2 Department of Biochemistry and Molecular Biology, School of Life Sciences, Peking University, Beijing, 100871 China

**Dear Editor,**

Acute promyelocytic leukemia (APL) caused by the malignant proliferation of bone marrow-derived cells, which is driven by the chromosomal translocation, leading to the fusion of the promyelocytic leukemia gene (*PML*) and the retinoic acid receptor-a gene (*RARA*) (de The et al., 1990; Goto et al., 2011). The oncogenic protein PML-RARA has been found specifically in more than 95% APL patients and is crucial for both the pathogenesis and therapeutics of APL (de The et al., 1990; Zhou et al., 2007; Wang and Chen, 2008). Arsenic trioxide (AS_2_O_3_), an ancient traditional Chinese medicine, has recently been adopted as the first-line treatment for APL patients by the National Comprehensive Cancer Network (Wang and Chen, 2008; Zhu and Huang, 2014), which greatly improved the 5-year overall survival of APL patients (Li et al., 2002; Zhou et al., 2007; Wang and Chen, 2008; Lo-Coco et al., 2013). Mounting biological studies revealed that AS_2_O_3_ cures APL by initiating PML-RARA degradation through specific binding to the PML moiety (Lallemand-Breitenbach et al., 2001; Zhang et al., 2010). Mechanistically, AS_2_O_3_ binds directly to the dicysteine residues C212/213 located in the B2 domain of PML, which induces PML oligomerization, NB formation, SUMOylation, and ultimate degradation of PML (Lallemand-Breitenbach et al., 2008; Jeanne et al., 2010; Zhang et al., 2010).

Unfortunately, although the application of AS_2_O_3_ dramatically promoted the clinical remission of APL patients, some patients still experienced a relapse with AS_2_O_3_ treatment in the clinical practice (Goto et al., 2011; Zhu et al., 2014). Therefore, characterizing the molecular mechanism of AS_2_O_3_ resistance is critical for optimizing the clinical application of AS_2_O_3_. Recently, it has been reported that 9 arsenic-resistant APL patients harboring PML-RARA mutations, including S214L, A216T, L217F and S220G which locate in the B2 domain of PML-RARA (Zhu et al., 2014). Among them, 8 patients could not be induced a second remission and died (Zhu et al., 2014). These clinical observations provided *in vivo* evidence that mutations in the hot-spot domain (C212-S220) of PML-RARA might contribute to the unresponsiveness to AS_2_O_3_ treatment, and the underlying molecular mechanism of PML-RARA mutations with AS_2_O_3_ resistance deserve further study.

In this study, we constructed the expression plasmids of PML-RARA-S214L, A216T, L217F and S220G that were clinically observed in arsenic-resistant APL patients (Fig. 1A). APL cell line NB4 cells were transfected with the indicated plasmid and a time-course study was performed with 2 µmol/L AS_2_O_3_. As shown in Fig. 1B, AS_2_O_3_ induced a significant degradation of wild-type PML-RARA. Similar results were observed with the mutants L217F and S220G. Conversely, addition of AS_2_O_3_ did not induce the degradation of S214L, even the treatment time was extended to 36 h. While in the case of A216T, the abundance of protein decreased moderately compared with wild-type PML-RARA. Quantification for the protein abundance of distinct PML-RARA mutants with AS_2_O_3_ treatment revealed that mutations occurred in different sites of PML-RARA B2 domain response distinctly to AS_2_O_3_ treatment (Fig. 1C). The unresponsiveness of mutant S214L to AS_2_O_3_ indicates the essential role of Ser214 amino residue in mediating AS_2_O_3_-induced physiological consequence.

**Figure 1:**
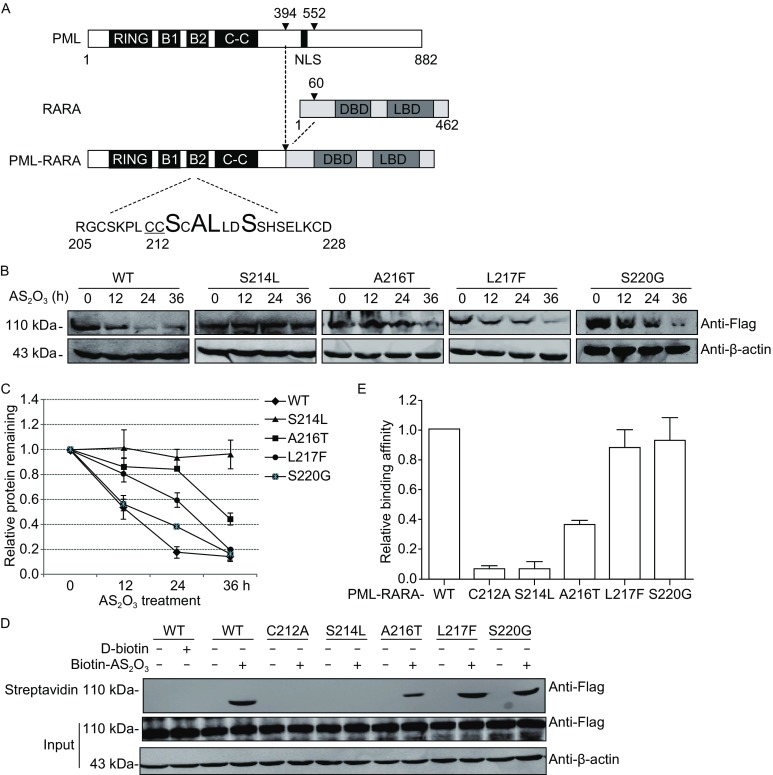
**The effects of AS**_**2**_ **O**_**3**_ **on the degradation of PML-RARA mutants**. (A) Schematic model of PML, RARA, and the fusion protein. The functional domains of PML and RARA are indicated with black and gray, respectively. RING refers to the RING finger; B1 and B2, B-box motifs; C-C, coiled-coil; and DBD, DNA-binding domain. Black arrows indicate the break points of the fusion proteins PML-RARA. The CC motif (C212/213) required for AS_2_O_3_ binding is underlined. The mutation sites Ser214, Ala216, Leu217 and Ser220 were highlighted in bold. (B) NB4 cells transfected with different plasmids were treated with 2 µmol/L AS_2_O_3_ for the indicated time and AS_2_O_3_-induced PML-RARA degradation was evaluated by Western blot using anti-Flag antibody. (C) Quantification of the relative protein level of PML-RARA mutants with AS_2_O_3_ treatment showed in Fig. 1B was calculated. The protein abundance of PML-RARA without AS_2_O_3_ treatment (0 h) was presented as ‘1’ and the level of PML-RARA with AS_2_O_3_ treatment for the indicated time was expressed relatively to this. The error bars represent standard error. (D) CHO cells transfected with indicated PML-RARA mutants were treated with 10 µmol/L AS_2_O_3_ for 2 h. Cells were harvested and lysed in 8 mol/L urea buffer. The supernatant was incubated with streptavidin agarose beads overnight at 4°C. After extensive wash, the streptavidin beads were resuspended in 30 µL SDS-PAGE loading buffer. The binding of arsenic with PML-RARA was analyzed by Western blot. (E) The binding affinity of PML-RARA mutants with arsenic observed in Fig. 1D was quantified. The ratio was described relatively to the binding affinity of wild-type PML-RARA with arsenic

AS_2_O_3_-induced degradation of PML-RARA is initiated by the direct binding between AS_2_O_3_ and PML-RARA (Jeanne et al., 2010). As different degradation pattern was observed above in the PML-RARA mutants, to detect the binding affinity of AS_2_O_3_ to different PML-RARA mutants, streptavidin agarose affinity assay was performed with *p*-aminophenylarsine oxide (PAPAO) as the probe (Zhang et al., 2010). PAPAO was conjugated to biotin, which was designed as Biotin-As. The result showed that biotin-As bound to PML-RARA, similar binding affinity was observed with PML-RARA-L217F and S220G (Fig. 1D and 1E). And the mutant A216T showed a relatively decreased binding affinity compared with the wild-type PML-RARA. Conversely, S214L mutation exhibited sharply diminished biotin-As binding (Fig. 1D and 1E), similar to the mutant C212A which has been demonstrated to mediate the direct arsenic binding (Jeanne et al., 2010). These results indicate that Ser214, together with the adjacent dicysteine motif C212/213, are equally essential in mediating the arsenic-PML binding, consistent with the data that S214L mutant is resistant to AS_2_O_3_-induced degradation.

Upon the binding of arsenic to PML-RARA, PML-RARA is subjected to hierarchical posttranslational modifications, such as SUMOylation, which is required for the responsiveness of PML-RARA to AS_2_O_3_ (Lallemand-Breitenbach et al., 2008; Jeanne et al., 2010; Zhang et al., 2010; Goto et al., 2011). Because distinct mutants of PML-RARA exhibited different AS_2_O_3_ binding affinity and degradation pattern followed by AS_2_O_3_ treatment, we next detected whether these mutants affect the basal SUMOylation of PML-RARA. As expected, SUMOylated PML bands were observed in cells transfected with wild-type PML-RARA. In contrast, the SUMOylation level of A216T elicited a decreased pattern, while mutation L217F and S220G showed no significant effect on PML-RARA SUMOylation. Notably, no SUMOylation band was observed for S214L (Fig. 2A). To confirm the data of S214L, the SUMOylated PML-RARA bands were detected with AS_2_O_3_ treatment. Compared with PML-RARA, SUMOylated S214L was not detected with or without AS_2_O_3_ treatment (Fig. 2B). The varying SUMOylation pattern of PML-RARA mutants is correlated with the degradation pattern of these mutants.

**Figure 2:**
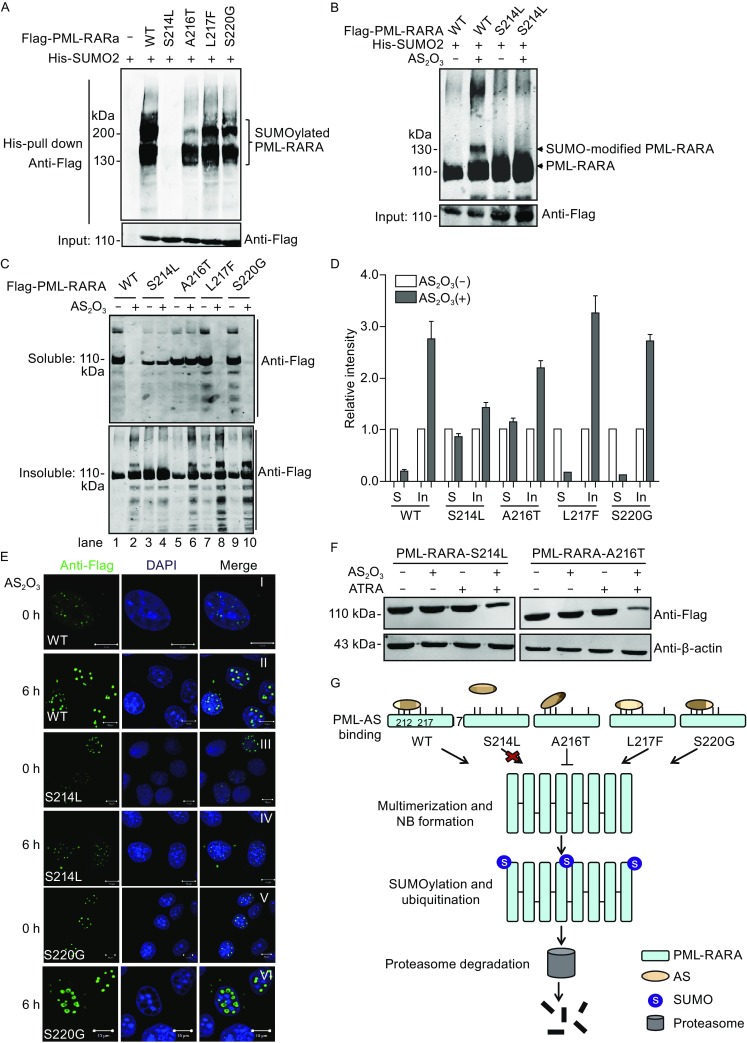
**Ser214 is required for the SUMOylation of PML-RARA**. (A) CHO cells were transfected with His-SUMO2 and PML-RARA or the mutants. At 36 h after transfection, cells were harvested and subjected to *in vivo* SUMOylation analysis. (B) 293T cells were transfected with the indicated plasmids and then cells were incubated with or without with AS_2_O_3_ for 4 h. (C) 293T cells harboring the expression plasmids of PML-RARA mutants were treated with or without 2 µmol/L AS_2_O_3_ for 4 h. Cells were harvested and lysed with RIPA buffer, and then the whole cell lysates were separated into soluble and insoluble fractions by centrifugation follow by immunoblotting. (D) The protein intensity of PML-RARA mutants in soluble (S) or insoluble (In) fraction was quantified. The ratio was expressed relatively to the sample without AS_2_O_3_ treatment. (E) HeLa cells transfected with flag-tagged PML-RARA or mutants S214L and S220G were treated with or without 10 µmol/L AS_2_O_3_ for 6 h. Anti-Flag antibody was used to detect the NB formation and the nuclear was stained with DAPI. Scale bar, 10 µm. (F) HeLa cells transfected with S214L or A216T expressing plasmids were treated with the drug (2 µmol/L, respectively) for 16 h. The abundance of S214L and A216T was detected by western blot with anti-Flag antibody. (G) On the basis of our and previous data, arsenic directly binds to the Cys212/213 and Ser214 amino acids in the PML B2 domain, leading to the formation of oligomerization and promote SUMOylation of PML, which followed by the ubiquitination and degradation of PML-RARA. Mutation of Ser214 totally disrupts the response of PML-RARA to AS_2_O_3_. A216T shows moderately defects in the response to AS_2_O_3_. L217F and S220G, mutations have no effect on the response of PML-RARA to AS_2_O_3_.

It has recently been demonstrated that AS_2_O_3_ exposure triggers the nucleoplasmic PML transfers toward the nuclear matrix and nuclear body (NB) (Jeanne et al., 2010; Goto et al., 2011). To detect the transfer of different PML-RARA mutants in response to AS_2_O_3_, we performed subcellular fraction assay using 293T cells that expressing wild-type PML-RARA or the mutants. As expected, in the presence of AS_2_O_3_, PML-RARA was observed mostly in the insoluble nuclear body fraction (Fig. 2C, lane 2). Similar results were detected with the mutants of L217F and S220G (Fig. 2C, lanes 8 and 10). Conversely, the transfer of S214L exhibited no response to AS_2_O_3_ treatment (Fig. 2C, lanes 3 and 4). The relative protein intensity in the soluble or insoluble fraction was quantified and depicted in Fig. 2D. It has been reported that PML-RARA forms multimerization via disulfide-mediated covalent binding in response to AS_2_O_3_, and subsequently triggers the NB formation (Jeanne et al., 2010). Given that the essential role of Ser214 in AS_2_O_3_ response, we performed immunofluorescence assay to detect the effect of mutation S214L on the subcellular localization of PML-RARA. HeLa cells were transfected with the indicated PML-RARA expression plasmids and treated with or without AS_2_O_3_. Wild-type PML-RARA and mutant S220G were detected in the nucleus as a microgranular pattern (Fig. 2E) and the localization was sharply altered to a macrogranular pattern in response to AS_2_O_3_ (Fig. 2E). Interestingly, S214L was localized diffusely in the nucleus in the presence or absence of AS_2_O_3_ (Fig. 2E), suggesting a non-responsiveness of S214L to AS_2_O_3_ treatment. Previous studies demonstrate that the combination of all-trans retinoic acid (ATRA) works better than AS_2_O_3_ alone in APL treatment. Therefore, we hypothesized that combining ATRA with AS_2_O_3_ might induce the degradation of S214L and A216T mutants. As expected, AS_2_O_3_-ATRA triggered the degradation of S214L and A216T (Fig. 2F). These results suggest that combination of AS_2_O_3_ with ATRA overcome the arsenic-resistance of PML-RARA mutations.

In this study, we revealed the molecular mechanism of the responsiveness of the recent clinically identified PML-RARA mutations to AS_2_O_3_ treatment. Among these mutants, S214L strikingly disrupted PML-arsenic binding, NB formation, basal SUMOylation, and degradation. Compared with S214L, mutation A216T showed moderate defect in AS_2_O_3_ response, while mutants L217F and S220G behave similarly to wild-type PML-RARA (Fig. 2G). These findings suggest distinct mutants of PML-RARA confer varying degree of AS_2_O_3_ resistance.

Results from GOTO et al. also reported the missense mutations of PML-RARA-A216V and L218P occurred in 2 patients with refractory/relapsed APL (Goto et al., 2011). These two mutations contribute to the aberrant PML-RARA SUMOylation and NB formation (Goto et al., 2011). When our work was underway, Liu et al. also demonstrated that PML-RARA S214L, A216T, L217F and S220G showed varying resistance to AS_2_O_3_ (Liu et al., 2016). In this study, we detected the direct binding of PML-RARA mutants with AS_2_O_3_. Mutant S214L exhibited no binding to AS_2_O_3_, demonstrating that S214, together with C212/213, is equally essential for mediating the effect of AS_2_O_3_. It is possible that mutations in these amino acid residues lead to a conformational change in PML structure, which disrupts the microenvironment for the direct PML-AS_2_O_3_ binding and abolishes the following physiological consequences including SUMOylaiton and degradation of PML-RARA. Further determination of the three dimensional structure of PML-RARA and PML-RARA-S214L are needed to elucidate the detailed molecular mechanism.

AS_2_O_3_ has been considered as a consolidation treatment for the relapsed APL patients, which has dramatically improved the event-free survival and overall survival in APL patients (Rice and de The, 2014). Mechanism study about the mutations in PML-RARA for AS_2_O_3_ resistance indicates that higher dosage of AS_2_O_3_ or the combination of AS_2_O_3_ with chemotherapy might be helpful for those patients harboring A216T mutation. The unresponsiveness of S214L to As_2_O_3_ exposure suggests that it is better to choose an alternative treatment strategy other than As_2_O_3_ in relapsed APL patients.

## Electronic supplementary material

Below is the link to the electronic supplementary material.

Supplementary material 1 (PDF 170 kb)
