# Supplementary material for: PML-RARA mutations confer varying arsenic trioxide resistance
Source: Protein Cell. 2016 Dec 27;8(4):296–301. doi: 10.1007/s13238-016-0356-4 (PMC5359182; doi:10.1007/s13238-016-0356-4)
Supplement: Supplementary file 1 — Supplementary material 1 (PDF 170 kb) [file 13238_2016_356_MOESM1_ESM.pdf]

MATERIALS AND METHODS

Cell culture

Human embryonic kidney 293T cells, HeLa cells and CHO cells were cultured in DMEM containing 10% fetal bovine serum (FBS, Gibco) at 37℃ in a 5% CO_2 humidified atmosphere. Human APL cell line NB4 cells were cultured in RPMI-1640 medium supplemented with 10% FBS.

Plasmids and antibodies

The expression plasmid of wild-type PML-RARA was constructed by inserting the cDNA of PML-RARA into the pcDNA-3Flag vector with designed primers (PML-RARA-forward: 5′-CggAATTCATggAgCCTgCACCCg, and reverse: 5′-CCgCTCgAgTCACggggAgTgggTggCCg). The mutation constructs of PML-RARA-S214L, A216T, L217F and S220G were generated by site-directed mutagenesis PCR with Taq Plus Master Mix (Vazyme Code: P212-03, Vazyme Biotech Co., Ltd, Nanjing, China) using the following primers:

S214L-forward: 5′-gCTgTgCTgCCTgTgCgCgCTCCTTgAC,

S214L-reverse: 5′-gTCAAggAgCgCgCACAggCAgCACAgC;

A216T-forward: 5′-CTgCTCgTgCACgCTCCTTgACAgCAgC,

A216T-reverse: 5′-gCTgCTgTCAAggAgCgTgCACgAgCAg;

L217F-forward: 5′-CgTgCgCgTTCCTTgACAgCAgC,

L217F-reverse: 5′-gCTgCTgTCAAggAACgCgCACg;

S220G-forward: 5′-gCTCCTTgACggCAgCCACAgTgAgCTC,

S220G-reverse: 5′-gAgCTCACTgTggCTgCCgTCAAggAgC, respectively.

The introduction of mutation was verified by DNA sequencing. Anti-Flag monoclonal antibody was purchased from Sigma (M2, Sigma-Aldrich (Shanghai) Trading Co., Ltd) and anti-β-actin Mouse Monoclonal antibody (BE0036-100) was from EASYBIO (Beijing, China).

In vivo SUMOylation assay

HEK293T or CHO cells were transfected with the indicated plasmids and the SUMOylation level of distinct PML-RARA mutants were detected by *in vivo* pull-down assay as previously described (Zhang et al., 2014).

Streptavidin agarose affinity assay

CHO cells transfected with wild-type or mutants of PML-RARA were treated with 10 μM Biotin-As for 2 h, and then cells were harvested and lysed in 8 M urea buffer (50 mM Tris-HCl, pH 8.0, 150 mM NaCl, 8 M urea). The supernatant was collected and incubated with streptavidin agarose beads (SA100-04, Life technology, 5791 Van Allen Way, Carlsbad, California, USA) overnight at 4 ℃. After washing four times with the urea buffer, the streptavidin beads were resuspended in 30 μl SDS-PAGE loading buffer. The binding of arsenic with PML was analyzed by western blot with indicated antibodies.

Subcellular Fraction

The subcellular fraction was performed as described previously (Goto et al., 2011). HEK293T cells were transfected with the indicated PML-RARA plasmids. At 24 h after transfection, cells were treated with 2 μM AS_2O_3 for 2 h and then lysed in RIPA buffer (50 mM Tris-HCl, pH 7.5, 150 mM NaCl, 0.5% sodium deoxycholate, 0.1% SDS, 1% NP-40, 0.2 mM PMSF, and EDTA-free protease inhibitor cocktail) and fractionated into supernatants and pellets by centrifugation at 12,000 rpm for 15 min. The soluble or insoluble fraction was collected to a new tube and equal volume loading buffer was added, respectively. Samples were boiled at 96 ℃ for 10 min and then analyzed by western blot with the indicated antibodies.

Immunofluorescence microscopy

The subcellular localization of PML-RARA or PML-RARA mutants in response to AS_2O_3 treatment was detected as previously described (Bai et al., 2014). Images were visualized with a confocal laser scanning microscope (Leica TCS SP5) and processed with the ZEN software. DAPI was used to stain the cell nuclei.

REFERENCES

Bai, D., Zhang, J., Xiao, W., and Zheng, X. (2014). Regulation of the HDM2-p53 pathway by ribosomal protein L6 in response to ribosomal stress. Nucleic Acids Res 42, 1799-1811.

Goto, E., Tomita, A., Hayakawa, F., Atsumi, A., Kiyoi, H., and Naoe, T. (2011). Missense mutations in PML-RARA are critical for the lack of responsiveness to

arsenic trioxide treatment. Blood 118, 1600-1609.

Zhang, J., Bai, D., Ma, X., Guan, J., and Zheng, X. (2014). hCINAP is a novel regulator of ribosomal protein-HDM2-p53 pathway by controlling NEDDylation of ribosomal protein S14. Oncogene 33, 246-254.
